# Defining a mechanistic link between pigment epithelium–derived factor, docosahexaenoic acid, and corneal nerve regeneration

**DOI:** 10.1074/jbc.M117.801472

**Published:** 2017-09-26

**Authors:** Thang Luong Pham, Jiucheng He, Azucena H. Kakazu, Bokkyoo Jun, Nicolas G. Bazan, Haydee E. P. Bazan

**Affiliations:** From the Department of Ophthalmology and Neuroscience Center of Excellence, School of Medicine, Louisiana State University Health New Orleans, New Orleans, Louisiana 70112-2223

**Keywords:** adipose triglyceride lipase, brain-derived neurotrophic factor (BDNF), cornea, lipid signaling, phospholipase A, DHA, NGF, PEDF, Sema7A, neuropeptides

## Abstract

The cornea is densely innervated to sustain the integrity of the ocular surface. Corneal nerve damage produced by aging, diabetes, refractive surgeries, and viral or bacterial infections impairs tear production, the blinking reflex, and epithelial wound healing, resulting in loss of transparency and vision. A combination of the known neuroprotective molecule, pigment epithelium–derived factor (PEDF) plus docosahexaenoic acid (DHA), has been shown to stimulate corneal nerve regeneration, but the mechanisms involved are unclear. Here, we sought to define the molecular events of this effect in an *in vivo* mouse injury model. We first confirmed that PEDF + DHA increased nerve regeneration in the mouse cornea. Treatment with PEDF activates the phospholipase A_2_ activity of the PEDF-receptor (PEDF-R) leading to the release of DHA; this free DHA led to enhanced docosanoid synthesis and induction of *bdnf, ngf*, and the axon growth promoter semaphorin 7a (*sema7a*), and as a consequence, their products appeared in the mouse tears. Surprisingly, corneal injury and treatment with PEDF + DHA induced transcription of neuropeptide y (*npy*), small proline-rich protein 1a (*sprr1a*), and vasoactive intestinal peptide (*vip*) in the trigeminal ganglia (TG). The PEDF-R inhibitor, atglistatin, blocked all of these changes in the cornea and TG. In conclusion, we uncovered here an active cornea–TG axis, driven by PEDF-R activation, that fosters axon outgrowth in the cornea.

## Introduction

The dense innervation of the cornea sustains the homeostatic integrity of the ocular surface. Damage to corneal nerves leads to a decrease in tear production and blinking reflex, as well as impaired epithelial wound healing, which results in loss of transparency and vision (1, 2). Many factors can alter corneal innervation, such as aging, diabetes, and viral and bacterial infections. Moreover, nerve damage occurs after refractive surgery, such as laser *in situ* keratomileusis (LASIK)^2^ and photorefractive keratectomy. This can diminish corneal sensitivity and, as a consequence, produce dry-eye disease that may cause neuropathic pain and ulcers and result in the need for corneal transplants (3). It takes 3–15 years to recover corneal nerves after LASIK (4, 5).

The glycoprotein pigment epithelium–derived factor (PEDF) has neuroprotective and antiangiogenic bioactivities (6). Our previous studies using a rabbit model of corneal injury have shown that upon activation of the PEDF receptor (PEDF-R) by the full-length PEDF (7) or its 44-mer neuroprotective domain (8) plus docosahexaenoic acid (DHA) corneal nerve regeneration is enhanced after injury (9–11). Concomitantly, synthesis of the docosanoid neuroprotectin D1 (NPD1) is enabled, which in turn increases nerve regrowth (12). The PEDF-R (13) is the product of a patatin-like phospholipase-2 (*pnpl2a*) gene and is also known as calcium-independent phospholipase A_2_ζ (iPLA2ζ) (14), adipose triglyceride lipase (15), and desnutrin (16). In this study, the term PEDF-R is used to describe this protein because the ligand is PEDF. This study is the first to report a function for PEDF-R in the innervation of peripheral nerves, particularly in the cornea. The PEDF-R is enriched in white and brown adipose tissues, which are innervated by sensory calcitonin gene-related peptide (CGRP)- and substance P (SP)-positive nerves (17, 18). However, the role of PEDF-R in the innervation of these tissues has not been explored.

The cornea is mostly innervated by trigeminal sensory fibers (19, 20). Although some sympathetic nerves are also present (2), the sensory neuropeptides CGRP and SP play key roles in the “trophic” efferent function of the cornea. Cornea–TG axis-inflammatory response has been observed after corneal injury (21), but its interactions and molecular mechanism are not well-understood. Moreover, neurotrophins are selected for transport to the sensory neurons, and retrograde transport is mediated by their receptors, the tropomyosin receptor kinases (Trks). Neurotrophins (22), semaphorin 7A (Sema7A) (23), and regeneration-associated genes (RAGs) are important for the axonal regeneration of peripheral nerves (24–26), and their expression has been reported in the cornea (27, 28). In this study, we focused on RAGs with transcriptional response in the TG neurons, which improve regeneration after injury (22–24).

Using a mouse corneal model that has an innervation similar to the human cornea (20), this work aimed to characterize the significance of the PEDF-R in regulating the molecular mechanism(s) activated in the cornea–TG axis that modulate nerve regeneration.

## Results

### Corneal nerve regeneration is enhanced by PEDF plus DHA in the mouse cornea

**Figure 1.**
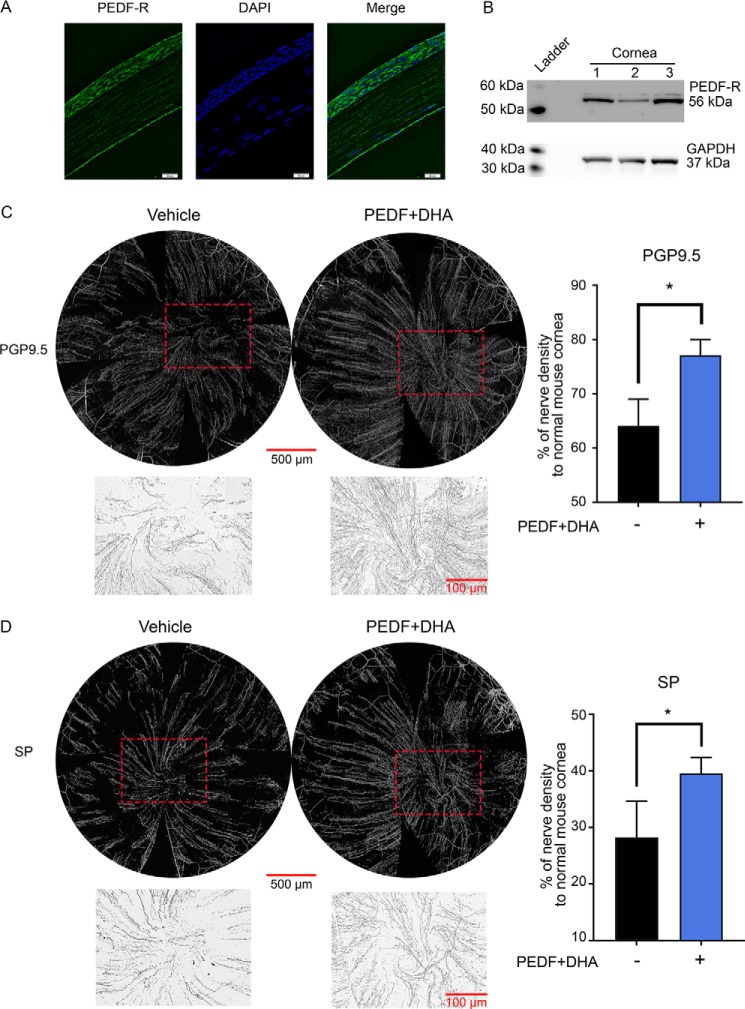
**PEDF + DHA enhances corneal nerve regeneration in the mouse.** *A,* representative image of a frozen section of a mouse cornea immunostained with anti-PEDR-R antibody (*green*) and DAPI (*blue*). *B,* expression of PEDF-R in mouse corneas (pool of six) by Western blotting. *C,* whole-mount images of corneal nerves were stained with anti-PGP9.5; *D,* anti-SP after injury and topical treatment with PEDF + DHA and vehicle three times a day for 7 days (Table 1 shows treatment concentrations). The *insets* in *C* and *D,* which are marked by a *dashed box* in the whole-mount images, show the amplified vortex area inverted to a *white background* in the treated corneas under ×10 objective lens. Data were normalized to the baseline (uninjured corneas). *Bars* represent average nerve density of four corneas ± S.D. *, *p* < 0.05 with *t* test statistical analysis to compare two groups at 95% of the confidence level. The experiment was repeated three times with similar results.

Fig. 1, *A* and *B*, shows that the PEDF-R is strongly expressed in the mouse corneal epithelium and endothelium (9). We then defined the effect of PEDF + DHA on corneal nerve regeneration after injury, as explained under “Experimental procedures,” using the concentrations described in Table 1. The treatment enhanced the density of protein gene product 9.5 (PGP9.5), a pan-neuronal marker, as well as sensory nerves positive to SP after 7 days of treatment (Fig. 1, *C* and *D*). Particularly, the PGP9.5-positive nerves regenerated faster in the EDF + DHA-treated group (77.1 ± 2.9% of the nerve density of non-injured corneas) in comparison with the vehicle-treated corneas (64.1 ± 4.9%, *p* < 0.05), whereas the density of SP-positive nerves were 39.5 ± 2.8 and 28.3 ± 6.4%, respectively.

**Table 1:** **Concentration of compounds used in different treatments** PEDF was purchased from Bio Products (Middletown, MD). DHA and atglistatin (a PEDF-R inhibitor) were purchased from Cayman Chemical (Ann Arbor, MI). The 44-mer PEDF peptide was prepared by GenWay Biotech, Inc. (San Diego). NPD1 was a kind gift from Dr. Nicos A. Petasis, Loker Hydrocarbon Research Institute, University of Southern California, Los Angeles.

Experiment (Figs.)	Compounds	Kind of treatment		
		Topical (10 μl/eye)	i.p.	*Ex vivo* organ culture
		*Ng*	μ*mol/kg*	
1–6	PEDF	0.5		50 ng/ml
1–6	DHA	100		50 nm
4	44-mer PEDF	0.05		5 ng/ml
4	NPD1	10		50 nm
5, 6	Atglistatin	23.7	200	

### Gene induction by PEDF + DHA in the injured corneal mouse model

**Figure 2.**
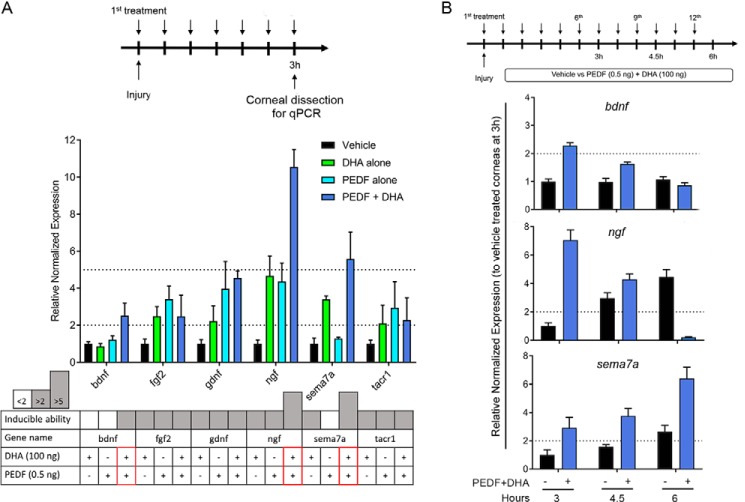
**Gene induction in the mouse cornea after injury and treatment with PEDF + DHA.** *A,* experimental design (*top*) and the induction of genes by DHA, PEDF, and PEDF + DHA, with the cutoff at a 2- and 5-fold increase (*p* < 0.05). In the *lower chart*, results are shown with three levels of gene induction: <2, between 2 and 5, and >5. *Red boxes* label three genes with higher expression by PEDF + DHA: *bdnf*, *ngf*, and *sema7a. B,* gene induction of *bdnf*, *ngf*, and *sema7a* by PEDF + DHA as a function of time. Mice were treated as described under “Experimental procedures” (*top*). Levels of transcripts were normalized to *actb*, *gapdh,* and *tbp* (primer sequences in Table 2). The *bars* represent the mean of three samples ± S.D. A pool of six corneas/sample was used for the gene expression study.

Our previous studies showed that a combination of PEDF + DHA is needed to obtain a significant stimulation in nerve regeneration (10). To decipher the gene targets engaged in PEDF + DHA-mediated nerve regeneration, we investigated the effect of PEDF, DHA, or the combination of PEDF + DHA in the induction of 10 genes, which included five induced genes (*bdnf*, *cntfr*, *fgf2*, *gdnf,* and *ngf*) selected from the screening of a panel of 62 genes (supplemental Table S1) as well as five additional genes (*cd40*, *npy*, *nrg1*, *sema7a,* and *tacr1*) chosen because previous studies demonstrated that they were related to nerve regeneration (22, 23, 29, 30). The primer sequences and complete names of these 10 genes are provided in Table 2. Injured mice received topical treatment of PEDF, DHA, and PEDF + DHA every 30 min for 3 h (Fig. 2*A*, *top*) before mouse corneas were analyzed by qPCR. The results showed that from the genes chosen, there were six genes induced by PEDF + DHA (Fig. 2*A*, *middle*). A simplified chart was used to display the three levels of gene induction, <2, >2, and >5 times, and to clearly observe the most significant changes in the treated conditions (PEDF + DHA, PEDF or DHA). Three potential genes (*bdnf*, *ngf*, and *sema7a*) were induced greater with PEDF + DHA than with either PEDF or DHA alone (Fig. 2*A*, *bottom*). The transcriptional induction of these three genes was further studied at 3, 4.5, and 6 h after injury and treatment with PEDF + DHA (Fig. 2*B*, *top*). There were three different time courses of gene induction: (i) *bdnf* was induced by PEDF + DHA treatment at 3 h and decreased to baseline levels at 6 h; (ii) *ngf* was induced early but at a lower amount than the vehicle at 6 h; and (iii) a constitutively elevated gene, *sema7a*, was induced at all time points with respect to the vehicle-treated group (Fig. 2*B*, *bottom*).

**Table 2:** **Primer sequences for gene expression analysis by q-PCR in the cornea** Three housekeeping genes, which were used to normalize the gene expression level, are shown in boldface in the table.

Gene	Name	Primer sequence	
		Forward	Reverse
*bdnf*	Brain-derived neurotrophic factor	TTGTTTTGTGCCGTTTACCA	GGTAAGAGAGCCAGCCACTG
*cd40*	CD40 molecule	TTGTTGACAGCGGTCCATCTA	GCCATCGTGGAGGTACTGTTT
*cntf*	Ciliary neurotrophic factor	TCTGTAGCCGCTCTATCTGG	GGTACACCATCCACTGAGTCAA
*fgf2*	Basic fibroblast growth factor	GCGACCCACACGTCAAACTA	TCCCTTGATAGACACAACTCCTC
*gdnf*	Glial cell-derived neurotrophic factor	TCTTTCGATATTGCAGCGGTT	GTCACTTGTTAGCCTTCTACTCC
*ngf*	Nerve growth factor	TTTGGAAACTCCTAGTGAACA	GTATAGAAAGCTGCGTCCTT
*npy*	Neuropeptide Y	ATGCTAGGTAACAAGCGAATGG	TGTCGCAGAGCGGAGTAGTAT
*nrg1*	Neuregulin 1	GAGGTGAGAACACCCAAGTCA	TGGTCCCAGTCGTGGATGTAG
*sema7a*	Semaphorin 7A	GGTGATGTCACTTGGTGAGATG	TCTTCCCGTTGTATTCCTGCT
*tacr1*	Tachykinin receptor 1	CTCCACCAACACTTCTGAGTC	TCACCACTGTATTGAATGCAGC
***actb***	**Actin β**	GTGACGTTGACATCCGTAAAGA	GCCGGACTCATCGTACTCC
***gapdh***	**Glyceraldehyde-3-phosphate dehydrogenase**	AGGTCGGTGTGAACGGATTTG	TGTAGACCATGTAGTTGAGGTCA
***tbp***	**TATA box-binding protein**	AGAACAATCCAGACTAGCAGCA	GGGAACTTCACATCACAGCTC

### Selective increase of neurotrophic factor signaling

**Figure 3.**
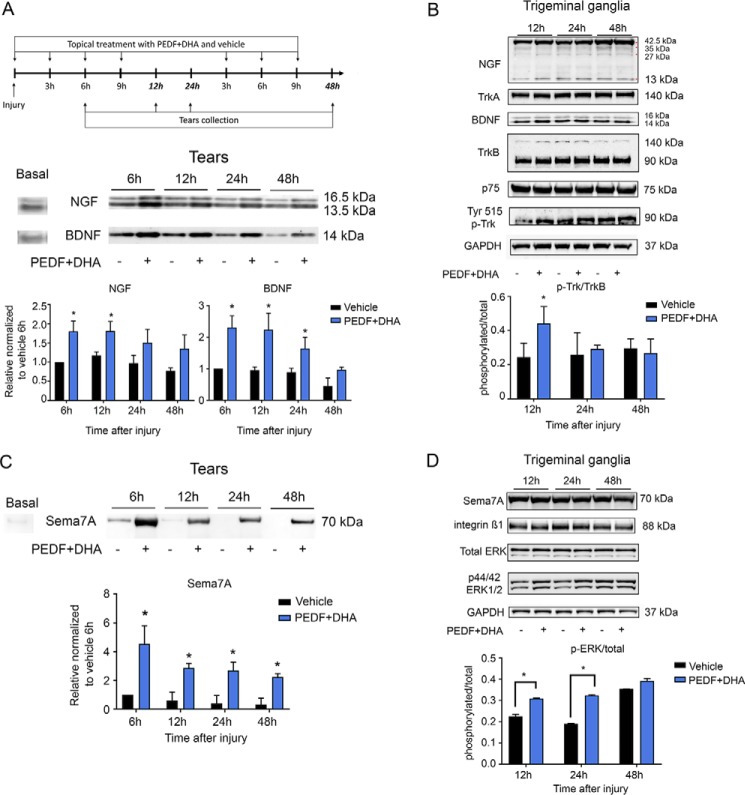
**PEDF + DHA treatment increases secretion of NGF, BDNF, and Sema7A in tears and the phosphorylation of TrkB and ERK1/2 in TG.** *A,* corneas were injured and treated with PEDF + DHA and tears collected as shown in the experimental design (*top*). Seven micrograms of protein from the mouse tear film (pool of six eyes/sample) were used for Western blot analysis of BDNF and NGF (*bottom*). *B,* Western blot analysis of TrkA, TrkB, p75, Tyr-phosphorylated Trks, and GAPDH in the TG (pool of six TGs, 50 μg of protein per well). *Bars* in *A* and *B* represent the mean of two experiments (two different pooled sample sets for each experiment, four samples in total) ± S.D. *, *p* < 0.05 with the *t* test analysis in comparison with the vehicle at the same time point. *C,* Western blot of Sema7A secreted to tears after PEDF + DHA treatment. Seven micrograms of protein collected from tears (pool of six eyes/sample) were used. *D,* Western blot analysis of intracellular integrin β1, total ERK, p44/p42 ERK1/2, and GAPDH in the TG (pool of six TGs, 50 μg of protein per well) of corneas treated with PEDF + DHA or vehicle. Details about the antibodies used are in Table 3. *Bars* in *C* and *D* represent the mean of two experiments (two different pooled sample sets for each experiment, four samples in total) ± S.D. of p-ERK/total ERK ratio. *, *p* < 0.05 with *t* test analysis in comparison with the vehicle at the same time points.

Levels of NGF and BDNF were analyzed by Western blotting using the antibodies as described (Table 3). Tears were collected before injury and at 6, 12, 24, and 48 h after injury and treatment with PEDF + DHA or vehicle, as explained under “Experimental procedures” (Fig. 3*A*). There were differences between the secretion of NGF and BDNF after treatment (Fig. 3*A*); although NGF secretion was increased significantly at 6 and 12 h, relative to vehicle-treated group, BDNF secretion was increased at 6, 12, and 24 h after injury and treatment (Fig. 3*A*).

**Table 3:** **Characteristics of the antibodies used in the study**

No.	Name	Company	Catalog no.	Type	Host	Dilution
1	BDNF	Santa Cruz Biotechnology	sc-546	Polyclonal	Rabbit	1:250
2	NGF	Santa Cruz Biotechnology	sc-549	Polyclonal	Rabbit	1:250
3	Sema7A	Santa Cruz Biotechnology	sc-135263	Polyclonal	Rabbit	1:250
4	TrkA	Abcam	ab76291	Monoclonal	Rabbit	1:500
5	TrkB	Cell Signaling	4603S	Monoclonal	Rabbit	1:500
6	p-Trk	Santa Cruz Biotechnology	sc-8058	Monoclonal	Mouse	1:500
7	p-75	Santa Cruz Biotechnology	sc-8317	Monoclonal	Rabbit	1:250
8	p-ERK	Cell Signaling	9106	Monoclonal	Mouse	1:500
9	Total ERK	Santa Cruz Biotechnology	sc-94	Monoclonal	Rabbit	1:500
10	Integrin	Santa Cruz Biotechnology	sc-8978	Polyclonal	Rabbit	1:250
11	ATGL	Cayman	10006409	Polyclonal	Rabbit	1:250
12	GAPDH	Santa Cruz Biotechnology	sc-25778	Polyclonal	Rabbit	1:500
13	PGP9.5	Abcam	ab108986	Monoclonal	Rabbit	1:1000
14	SP	Santa Cruz Biotechnology	sc-21715	Monoclonal	Rat	1:200
15	SPRR1A	Abcam	ab125374	Polyclonal	Rabbit	1:1000

Most of the nerve terminals in the corneal epithelium derived from neurons originated in the TG (20). To investigate whether there is an interaction between the cornea and TG after PEDF + DHA treatment, corneas and TG from the same mice under the same conditions were pooled and analyzed, respectively, by Western blotting. There were no changes in the levels of BDNF, NGF, and Sema7A in the corneas (data not shown). In the TG, NGF was detected as a strong band of the pro-form (42.5–28 kDa) and as a weak band of the mature form (13.5 kDa) (31); BDNF, however, was detected mainly as the mature form (14–16 kDa). No differences in their protein levels were found in the presence of PEDF + DHA, relative to the vehicle-treated groups (Fig. 3*B*). In addition, the neurotrophin receptors TrkA and TrkB were analyzed. The glycosylated form of TrkA was detected as a band at 140 kDa (32), whereas the TrkB receptor showed two bands at 140 and 90 kDa, which are considered the glycosylated and the full mature forms, respectively (33). The p75 co-receptor of TrkA and TrkB was also expressed. However, there were no differences in the expression of these receptors between the PEDF + DHA- and vehicle-treated groups (Fig. 3*B*). Because our results showed that BDNF was increased in mouse tears up to 24 h by PEDF + DHA (Fig. 3*A*), we hypothesized that the treatment might alter the phosphorylation of the Trk receptors in the TG. A phosphorylated Trk (p-Trk) antibody, which detects Tyr-496 p-TrkA and Tyr-515 p-TrkB in the mouse, was used. Only a p-TrkB band (90 kDa), which increased at 12 h after injury and treatment with PEDF + DHA, compared with vehicle-treated group, was found (Fig. 3*B* and supplemental Fig. S1). This phosphorylation corresponds to the 515 tyrosine residues in TrkB.

### Activation of Sema7A signaling

Sema7A belongs to a family of proteins that are expressed in several tissues, including the nervous system. *In vivo*, Sema7A is present as a glycosylphosphatidylinositol (GPI)-anchored protein that lacks the cytoplasmic domain and can be secreted into cerebrospinal fluid (34). Sema7A stimulates axon growth of the central and peripheral nervous system (23, 35). Because PEDF + DHA treatment increases the gene expression of *sema7a* (Fig. 2, *B* and *C*), the protein levels of Sema7A were analyzed in cornea samples at 12, 24, and 48 h after injury and treatment. There were no differences between PEDF + DHA- and vehicle-treated groups (data not shown). However, an increase in Sema7A expression was found in the tears (Fig. 3*C*) up to 48 h. This increase in secreted Sema7A in tears after PEDF + DHA treatment was correlated with the transcriptional induction of this gene in the cornea. The vehicle-treated corneas showed a small amount of Sema7A secretion at 6 h after injury that was similar to basal levels (before injury), and this amount decreased over time after the injury occurred.

Sema7A has been reported to act through integrin receptors and mitogen-activated protein kinase signaling (23). To investigate the cornea–TG interaction, we analyzed the signaling components in the TG. Phosphorylation of ERK1/2 was increased in the TG after corneal treatment with PEDF + DHA at 6, 12, and 24 h (the integrin β1 receptor and endogenous Sema7A were not changed; Fig. 3*D* and supplemental Fig. S1).

### Involvement of 44-mer PEDF and NPD1 signaling

**Figure 4.**
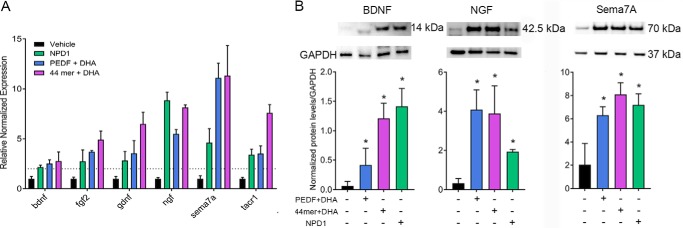
**Stimulation of gene and protein expression of neurotrophins and Sema7A by 44-mer PEDF + DHA, NPD1, and PEDF + DHA.** *A,* qPCR gene expression analysis of mouse corneas at 3 h after injury and treatments. The concentrations of the compounds are described in Table 1. All genes were significantly induced in comparison with the vehicle-treated groups (six corneas/sample, *p* < 0.05). Levels of transcripts were normalized to *gapdh*, *tubb3,* and *tbp* (primer sequences in Table 4). *B,* Western blot analysis of secreted BDNF, NGF, and Sema7A into the media of injured corneas in organ culture treated with the compounds or vehicle for 24 h (six corneas/sample). The data were normalized to GAPDH as an internal standard. *, *p* < 0.05 with *t* test analysis in comparison with the vehicle. The *bars* represent the mean of three samples ± S.D. The experiment was repeated once with similar results.

Our previous studies have shown that the 44-mer neuroprotective domain of PEDF targeted the PEDF-R and accelerated nerve regeneration in a rabbit model (9); NPD1, which was synthesized by the PEDF + DHA treatment, also increased corneal innervation after injury (10–12). To determine whether these compounds could induce the same genes as PEDF + DHA, we treated the injured mouse corneas with 44-mer PEDF + DHA at a similar molar level to full-length PEDF + DHA or NPD1 (the concentrations are described in Table 1) and compared our results with those genes obtained from animals treated with PEDF + DHA (Fig. 4*A*). Both NPD1 and 44-mer PEDF + DHA induced the six genes that we observed previously (Fig. 2*A*), and no significant differences were recorded among the PEDF + DHA, 44-mer PEDF + DHA, and NPD1 treatments while using the paired *t* test and one-way ANOVA.

It is important to note that our experiments do show that PEDF + DHA increase the secretion of NGF, BDNF, and Sema7A in tears (Fig. 3, *A* and *C*). The origin of these proteins, however, are not completely defined because the tear components are influenced not only by the cornea but also by the lacrimal and meibomian glands and conjunctiva. We measured the protein levels of BDNF, NGF, and Sema7A, which were stimulated with PEDF + DHA, 44-mer PEDF + DHA, or NPD1, using the corneal *ex vivo* organ culture to ensure that those proteins were secreted only by the cornea without input from other organs. After 24 h of organ culture, significant amounts of BDNF, NGF, and Sema7A were secreted to the media when the injured corneas were stimulated with the compounds, as compared with the vehicle-treated animals (Fig. 4*B*). Interestingly, in this *ex vivo* culture, NGF was secreted as a pro-form NGF (42.5 kDa), although its mature form (13.5 kDa) was detected in the tears. In contrast, the same forms of BDNF and Sema7A were detected as 14- and 70-kDa bands, respectively, similar to the *in vivo* studies (Figs. 3, *A* and *C*, and 4*B*).

### PEDF-R promotes an interaction between corneal lipid signaling and TG transcriptional activation

**Figure 5.**
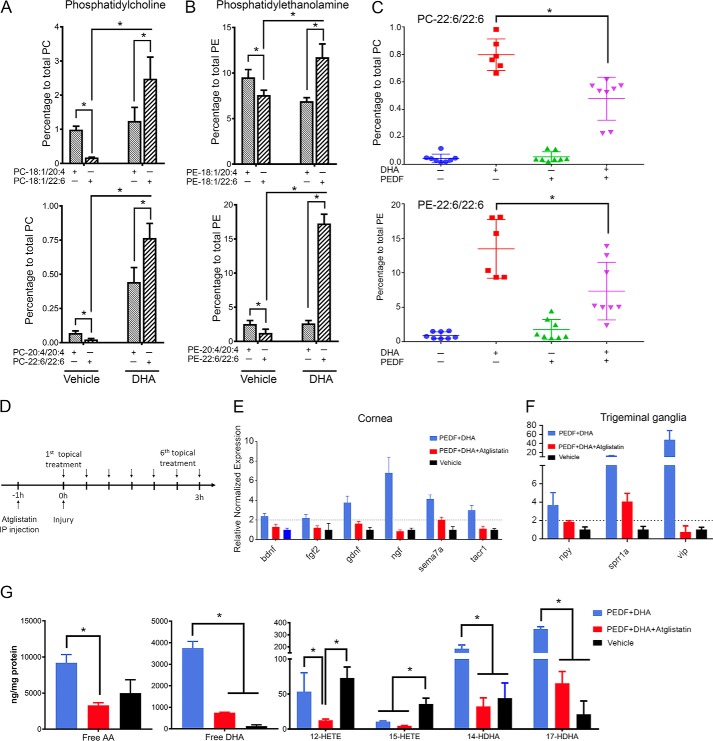
**DHA incorporation in corneal PC and PE molecular species, from basal to treated conditions.** Effect of atglistatin on gene expression, release of DHA and AA, and synthesis of hydroxy-derivatives was examined. *A* and *B,* comparison of different molecular species of PC and PE in injured corneas in mice after 1 h of topical DHA treatment *in vivo* analyzed by LC-MS/MS. Each plot shows the percentage of specific PC/PE species in the vehicle- (*left*) and DHA (*right*)-treated corneas. Data were collected from eight corneas individually (one cornea/sample). *, *p* < 0.05 with the *t* test statistical analysis to compare two groups at 95% of the confidence level. *C,* quantification of 22:6/22:6-containing PCs and PEs in the presence of PEDF, DHA, and PEDF + DHA. Single data point represents one treated cornea. *, *p* < 0.05 with ANOVA analysis plus Fisher post hoc test at 95% of the confidence level. *D,* experimental design of mice treated with atglistatin injected (i.p.) before injury and topically treated with PEDF + DHA, PEDF + DHA + atglistatin, or vehicle after injury. *E* and *F*, gene expression analysis in corneas (*E*) and TG (*F*) at 3 h after injury and treatment. *G,* mass spectrometry-based lipidomic analysis of mouse corneas as explained under “Experimental procedures.” *, *p* < 0.05 with ANOVA analysis plus Fisher post hoc test at 95% of the confidence level. For lipid analysis, a pool of six corneas/sample was used. *Bars* represent the mean of three experiments ± S.D.

Our earlier studies have found that in rabbit corneas, the concentration of DHA in membrane lipids is very low (36). We also found that mouse cornea contains more arachidonic acid (AA, 20:4) than DHA in phosphatidylcholine (PC), and the TG has a higher amount of DHA than AA (data not shown). Therefore, the addition of DHA to the treated corneas is very important for increasing the formation of docosanoids. Phosphatidylcholine and phosphatidylethanolamine (PE) are the main membrane phospholipids in the cornea (36, 37). To determine whether the added DHA was incorporated into the membrane phospholipids, the composition of PC and PE molecular species that contain the biologically-active fatty acids AA and DHA (22:6) were analyzed by liquid chromatography-tandem mass spectrometry (LC-MS/MS) after corneal injury and treatment with DHA or vehicle for 1 h. We found that the PE species incorporates DHA in a much higher proportion than PC (Fig. 5, *A* and *B*). There was a 14-fold increase of the PC species containing oleic acid (18:1) at the *sn*-1 and DHA at the *sn*-2 position after 1 h of topical DHA treatment, in comparison with vehicle treatment (Fig. 5*A*, *top*). Interestingly, these molecular changes were more pronounced in the PC with two DHAs in the C1 and C2 position of glycerol, with a 32-fold increase over the vehicle-treated corneas. The two PEs (18:1/22:6 and 22:6/22:6) showed an increase of DHA of 1.5 and 14 times, respectively, compared with the vehicle-treated group. Next, we targeted the quantification of the di-DHA-containing PC and PE species in corneas treated with vehicle, DHA, PEDF, or PEDF + DHA. The individual plots showed a trace amount of these PC/PE in the vehicle and PEDF-treated corneas (Fig. 5*C*) and a significant reduction of di-DHA-containing PCs and PEs in the PEDF + DHA-treated corneas, in comparison with the DHA-treated group. This indicates that PEDF, by activating its PEDF-R, released free DHA from the PC and PE pools.

To understand the role of lipid signaling when the PEDF-R is activated, we used atglistatin, an inhibitor of PEDF-R (concentration in Table 1) (38). An intraperitoneal (i.p.) injection of the inhibitor was applied 1 h before injury, and treatment was followed by topical application of the inhibitor after injury every 30 min for 3 h (Fig. 5*D*). In preliminary experiments, a combination of i.p. injection and topical treatment of the inhibitor produced stronger inhibition than topical treatment alone. Complete inhibition of the induction of the six genes by PEDF + DHA occurred when corneas were treated with the inhibitor (Fig. 5*E*). Because the cornea is devoid of blood vessels, we could not explain what effect the i.p. injection of the inhibitor had on corneal gene expression. One possibility is that the TG might play a role in the mechanism. Therefore, we analyzed whether there were any changes in the gene expression of neuropeptides and receptors in the TG of the same treated animals. For these studies, we chose 14 genes whose primer sequences are shown in Table 4. We focused on four of the most abundant neuropeptides in TG (CGRP, NPY, SP, and VIP) and their receptors, as well as three receptors for NGF and BDNF (TrkA, TrkB, and NGF receptor) and one RAG, small proline-rich repeated protein 1A (*sprr1a*), which is expressed in mouse sensory neurons and the spinal cord after injury (24). There were three TG genes (*npy*, *sprr1a*, and *vip*) that were induced by corneal treatment with PEDF + DHA (Fig. 5*F*) and that were inhibited by atglistatin. These results suggest that there is an interaction between the cornea and TG activated by PEDF-R that induced specific genes in each tissue.

**Table 4:** **Primer sequences for gene expression analysis by qPCR in the trigeminal ganglia** Three housekeeping genes, which were used to normalize the gene expression level, are shown in boldface in the table.

Gene	Name	Primer sequence	
		Forward	Reverse
*calca*	Calcitonin-related polypeptide α	CAGTGCCTTTGAGGTCAATCT	CCAGCAGGCGAACTTCTTCTT
*calclr*	Calcitonin gene-related peptide receptors	ATCTCAGCAGAGTCGGAAGAA	CAGGTCCTATTGCAGTAAAGGC
*ngfr*	Nerve growth factor receptor	CTAGGGGTGTCCTTTGGAGGT	CAGGGTTCACACACGGTCT
*npy*	Neuropeptide Y	ATGCTAGGTAACAAGCGAATGG	TGTCGCAGAGCGGAGTAGTAT
*npy1r*	Neuropeptide Y receptor 1	TGATCTCCACCTGCGTCAAC	ATGGCTATGGTCTCGTAGTCAT
*npy2r*	Neuropeptide Y receptor 2	TCCGGGAATACTCCCTGATTG	GCAAAACGTACAGGATGAGCAG
*npy5r*	Neuropeptide Y receptor 5	TTTGTCACGGAGAACAATACTGC	TGCGCTTTTTCATAACAGCCAT
*sprr1a*	Small proline-rich protein 1A	TTGTGCCCCCAAAACCAAG	GGCTCTGGTGCCTTAGGTTG
*tac1*	Tachykinin precursor 1	ATTCCTTTGTTGGACTAATGGGC	ACGTCTTCTTTCGTAGTTCTGC
*tacr1*	Tachykinin receptor 1	CTCCACCAACACTTCTGAGTC	TCACCACTGTATTGAATGCAGC
*trka*	Neurotrophic receptor tyrosine kinase 1	CAGTCTGATGACTTCGTTGATGC	CTCTTCACGATGGTTAGGCTTC
*trkb*	Neurotrophic receptor tyrosine kinase 2	GTTGACCCGGAGAACATCACG	ACTTTAAGCCGGAATCCACAAT
*vip*	Vasoactive intestinal peptide	AGTGTGCTGTTCTCTCAGTCG	GCCATTTTCTGCTAAGGGATTCT
*vipr2*	Vasoactive intestinal peptide receptor 2	AGGCCATTTATACCTTGGGCT	GCAGTAGACCTGAGCTGGAGTA
***tubb3***	**Tubulin β-III**	CGCACGACATCTAGGACTGA	TGAGGCCTCCTCTCACAAGT
***gapdh***	**Glyceraldehyde-3-phosphate dehydrogenase**	AGGTCGGTGTGAACGGATTTG	TGTAGACCATGTAGTTGAGGTCA
***tbp***	**TATA box-binding protein**	AGAACAATCCAGACTAGCAGCA	GGGAACTTCACATCACAGCTC

It is important to note that our experiments do show that exogenous DHA can incorporate into membrane PCs and PEs, and the bioactivity of PEDF, by activating PEDF-R, reduces the DHA-containing PC and PE species. To investigate the ability of PEDF-R to release the biologically-active fatty acids DHA and AA, we followed the experimental design described in Fig. 5*D*. After 3 h, corneas were harvested, and the release of DHA and AA as well as the synthesis of their hydroxy-derivatives were analyzed by tandem LC-MS/MS as described under “Experimental procedures.” The amount of both free AA and DHA was increased by PEDF + DHA, and these fatty acids were inhibited by atglistatin (Fig. 5*G*). In comparison with vehicle-treated corneas, PEDF + DHA increased the release of DHA ∼30-fold, although the release of free AA was increased 2-fold. Importantly, there was a 5× inhibition of released DHA when corneas were treated with PEDF + DHA + atglistatin (Fig. 5*G*). This result shows an equivalence of the exogenous DHA incorporation in the membrane and the unesterified-DHA in the cornea because the vehicle-treated corneas (which lacked DHA supplementation) released a trace amount of free DHA. Previous studies have shown that DHA is metabolized to its 14- and 17- (*S*)-hydroperoxy-DHA (HpDHA) derivatives in the maresin and NPD1 pathways, respectively (39, 40). Both of these DHA derivatives were converted to the more stable forms as 14- and 17-hydroxyl-DHA (HDHA) and were stimulated by PEDF + DHA. In comparison with the vehicle-treated group, PEDF + DHA induced a 4.2 and 17.1 times higher formation of 14- and 17-HDHA, respectively, whereas atglistatin inhibited the PEDF + DHA-induced synthesis of 14- and 17-HDHA (Fig. 5*G*). Stimulation with PEDF + DHA produced lower amounts of 12- and 15-hydroxyeicosatetraenoic acids (HETEs) compared with the formation of DHA derivatives (Fig. 5*E*). The DHA derivative NPD1, which plays a central role in the working mechanism of PEDF + DHA, was not detected at 3 h after injury and treatment (10). Our previous experiments in rabbits, however, have shown that NPD1 increases in injured corneas after 1 and 2 weeks of treatment with PEDF + DHA (10).

### Lipid-signaling inhibition attenuates corneal nerve regeneration

**Figure 6.**
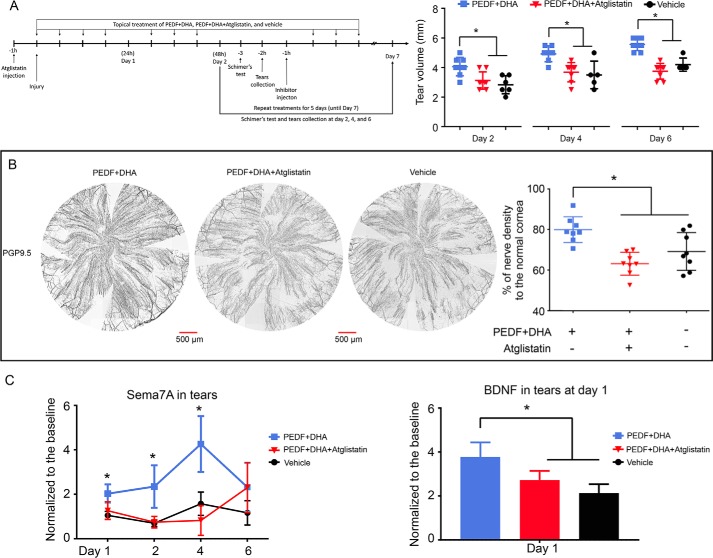
**Inhibition of PEDF-R decreases corneal nerve regeneration.** *A,* experimental design and Schirmer's test analysis. Volume of mouse tears was measured on days 2, 4, and 6 after injury and treatment. *, *p* < 0.05 with ANOVA plus Fisher post hoc test at 95% of confidence level. *B,* representative whole-mount images of corneal nerves stained with anti PGP9.5 and percent of nerve density compared with normal corneas after 7 days of treatment. *, *p* < 0.05 with ANOVA plus Fisher post hoc test at 95% of confidence level. *C,* effect of atglistatin on the levels of Sema7A and BDNF in tears analyzed by Western blotting (pool sample of six eyes, 7 μg of protein per well). The intensity of immunoreactive bands was normalized to the baseline (uninjured corneas). The *bars* represent the mean of three experiments ± S.D. *, *p* < 0.05 with ANOVA plus Fisher post hoc test at 95% of confidence level.

Finally, to demonstrate the role of PEDF-R in PEDF + DHA-stimulated corneal nerve regeneration, we treated injured mice for 7 days as explained under “Experimental procedures” (Fig. 6*A*). Alteration in corneal innervation decreased tear production, resulting in dry eye (41). Tear volume was measured by Schirmer's test at days 2, 4, and 6 after injury and treatment. At days 2 and 4, the average tear volume of corneas treated with PEDF + DHA was 4.06 and 4.93 mm, respectively, and was significantly higher than vehicle- and atglistatin-treated groups. On day 6, the tear volume peaked at 5.56 mm, whereas the PEDF + DHA + atglistatin administration showed an average value of 3.75 mm. Fig. 6*B* shows a representative image of corneal nerves as well as the percentage of nerve recovery after 7 days of injury and treatments. As already shown in Fig. 1*C*, nerve density after 7 days of treatment with PEDF + DHA increased to about 80% of the density for the uninjured corneas. There was significant inhibition of nerve regeneration when animals were treated with atglistatin, with a nerve density of 63.1% of the density for the uninjured corneas (*p* < 0.05).

We also investigated the contribution of PEDF-R activity to the tear secretion of Sema7A and BDNF (Fig. 6*C*). In agreement with our previous results (Fig. 3*A*), BDNF secretion showed an increase only at day 1, and atglistatin significantly decreased secretion of the neurotrophin. Sema7A secretion in the mouse tears, however, was increased for the first 4 days of treatment with PEDF + DHA, and atglistatin inhibited the effect of PEDF + DHA at days 1, 2, and 4.

## Discussion

In these experiments, our goal was to determine the molecular events of PEDF plus DHA on stimulating corneal nerve regeneration in an *in vivo* mouse injury model. Using a model that damages stromal nerves, this study showed that the density of both the total nerves stained with PGP9.5 antibody and the SP-positive nerves increases after 1 week of PEDF + DHA stimulation (Fig. 1*C*). Furthermore, similar results were observed recently in a diabetic mouse model (42).

One interesting finding was that regeneration of the SP-positive nerves was slower than regeneration of the total nerves stained with the PGP9.5 antibody. As is the case with other neurotransmitters, SP is synthesized in the cell body and then transported along the tubulin to the axon terminal (43). One possibility is that after injury, the SP content is released in the peripheral terminals of the nociceptive fibers to stimulate cell migration (44), and as a result, this delays the recovery of the SP-positive fibers. This is in agreement with our previous studies in rabbits in which, after 8 weeks of lamellar keratectomy and treatment with PEDF + DHA, we found a strong SP-positive staining in the epithelium (11).

**Figure 7.**
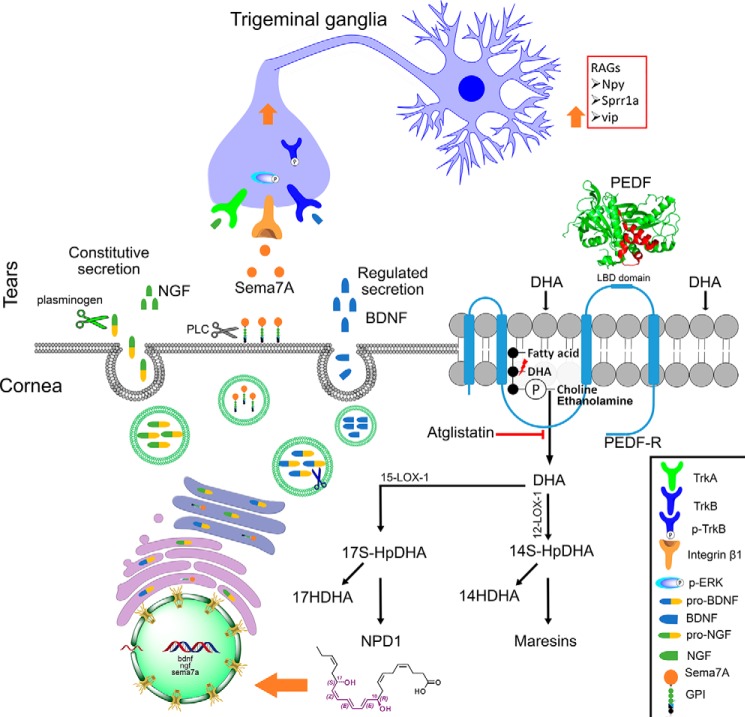
**Working model of the action of PEDF + DHA in enhancing corneal nerve regeneration.** PEDF via its 44-mer neuroprotective domain (in *red*) activates PEDF-R in the cornea (amplify for clarification). This transmembrane receptor with iPLA_2_ activity released DHA, enriched in the *sn*-2 position of membrane phospholipids, by DHA supplementation. Mouse corneas express a 12- and 15-lipoxygenase (*LOX*) (55); DHA is converted to 17-HpDHA on the pathway to NPD1 by 15-LOX-1, and DHA is also converted to 14-HpDHA on the pathway to maresin-1 by 12-LOX-1. Docosanoids such as NPD1 (and possibly others not yet identified) induce the gene expression and protein levels of the neurotrophins NGF, BDNF, and Sema7A (all of which are secreted to tears), and of RAGs *vip, npy*, and *sprr1a* in the TG. Phosphorylation of TrkB and ERK 1/2 occurs in the TG as a result of BDNF and Sema7A secretion in the tear film. Inhibition of the phospholipase activity of the PEDF-R abolishes this signaling mechanism and corneal nerve regeneration.

Previous work has shown that both PEDF and DHA are required to stimulate nerve regeneration (10). The combined treatment induced a higher expression of *bdnf*, *ngf*, and *sema7a* than the expression produced by PEDF or DHA alone (Fig. 2*A*). Moreover, a similar induction occurred when corneas were treated with the neuroprotective 44-mer PEDF + DHA and with NPD1 (Fig. 4*A*), two compounds that we previously showed to increase corneal nerve regeneration (9). After injury and treatment with PEDF + DHA, the mature NGF and BDNF were secreted in tears. In addition, the phosphorylated TrkB in the TG indicates an activation of BDNF signaling in the TG that could influence neurite outgrowth in the cornea. In the mouse, activation of *bdnf* and *ngf* genes has been reported after lamellar keratectomy (22, 28), and our previous studies have shown the potential activity of NGF + DHA in accelerating corneal nerve outgrowth (45). Moreover, the action of PEDF in up-regulating the expression of *ngf* and *bdnf* has been shown in cerebellar granule neurons (46). Interestingly, NGF was detected as a 42.5-kDa pro-NGF in the cornea (data not shown) as well as in the media from *ex vivo* cultured corneas (Fig. 4*B*). Pro-NGF is cleaved by plasminogen, an enzyme expressed in tears (47), where two bands of mature NGFs (13.5 and 16.5 kDa) were found (Fig. 3*A*). In contrast, BDNF is synthesized in the endoplasmic reticulum as a 28–32-kDa pro-BDNF and moves through the Golgi apparatus to the trans-Golgi network, where distinct protein convertases and secretory vesicles of the regulatory pathway cleaved off the amino-terminal pro-domain of pro-BDNF to yield mature BDNF (14 kDa) (48). Despite the different secretion mechanisms (regulated secretion for BDNF *versus* constitutive secretion for NGF), both mature forms of NGF and BDNF were secreted into the tears (Fig. 7) and could support the axon outgrowth efficiently (22).

PEDF + DHA also stimulated the expression of Sema7A at transcriptional and translational levels and its secretion to tears (Figs. 2*A* and 3*C*). To our knowledge, this is the first report of the presence of Sema7A in tears. A previous study showed that *sema7a* mRNA increases in the injured mouse cornea, but protein expression does not increase (35). Coinciding with these previous findings, we did not detect differences in the translated Sema7A between the PEDF + DHA- and vehicle-treated corneas (data not shown), despite the strong transcriptional induction by PEDF + DHA. Sema7A is a GPI-anchored membrane-associated protein. The GPI is tagged to the carboxyl terminus of Sema7A and supports the attachment of Sema7A to the membrane (49). Cleavage of GPI by phospholipase C (PLC) may result in the release of Sema7A from the cell membranes (Fig. 7). PLC is expressed in the corneal epithelium and tears (50), and we found that Sema7A was released in the tear film as a 70-kDa protein that is known to activate MAPK signaling pathways (23). Activation of ERK1/2 in the TG occurs after injury and treatment. pERK1/2 phosphorylates c-Jun (51) and STAT3 (52), which are two transcriptional factors that stimulate the expression of specific RAGs such as *vip*, *npy*, and *sprr1a* (24–26). We show here that the expression of these particular genes was up-regulated by PEDF + DHA in the TG (Figs. 5 and 7). These results suggest that PEDF + DHA exerts an effect on TG signaling, stimulating the phosphorylation of TrkB and ERK1/2 that correlates with the activation of their ligands (BDNF and Sema7A) in the cornea.

PEDF-R shows transacylase, phospholipase, and triglyceride lipase activities (14). Triglyceride hydrolysis has been well-described because the PEDF-R deficiency mice are obese and contain enlarged adipose fat depots (53). Our results suggest that, in the cornea, the phospholipase activity of PEDF-R releases DHA because this polyunsaturated fatty acid is incorporated mainly in the *sn*-2 position of phospholipids, of which PCs and PEs are the most abundant (54). In addition, the cornea is devoid of blood vessels and therefore cannot be supplied with triglyceride, which is normally transported by plasma. We hypothesized that adding DHA shifts the lipid-related response from the eicosanoids to docosanoids in the cornea. This hypothesis was supported by incorporation of DHA in the PCs and PEs (Fig. 5, *A* and *B*) and by the significant reduction in 22:6/22:6 PC and PE molecular species when corneas were treated with PEDF + DHA (Fig. 5*C*). In fact, our lipidomics analyses show that PEDF + DHA produces lower levels of 12- and 15-lipoxygenase-1-related eicosanoids, as opposed to docosanoids (Fig. 5*G*). In addition, *in vivo* treatment with atglistatin, a potent, selective, and competitive inhibitor of PEDF-R (38), decreased the release of DHA from the mouse cornea and abrogated the synthesis of 14- and 17-HDHA. This action compromised the expression of PEDF + DHA-induced genes in both the cornea and TG (Figs. 5 and 7) and contributed to the slow regeneration of corneal nerves at day 7 after injury and treatment (Fig. 6). In summary, binding of PEDF to the receptor increases its phospholipase enzymatic activity 5–6-fold (7) and stimulates a signaling cascade that involves the following: 1) docosanoids; 2) gene and protein expression of NGF, BDNF, and Sema7A; 3) induction of RAGs genes *vip*, *npy,* and *sprr1a* in the TG; and, as a consequence, 4) increased corneal nerve regeneration (Fig. 7). Future studies using a genetic ablation PEDF-R model are warranted to further support the proposed mechanism of the PEDF + DHA action on corneal nerve regeneration.

Furthermore, the corneal injury provides a good model for understanding the mechanism of nerve regeneration in the peripheral nervous system. Using this model, one can assess the corneal nerve density (anatomy) and tear volume components (behaviors and responses), as well as the systemic interaction of the cornea–TG axis. In summary, by taking advantage of this useful model, we uncovered here an active cornea–TG axis, driven by PEDF-R activation, that fosters axon outgrowth in the cornea.

## Experimental procedures

### Animals

Eight-week-old male C57BL/6 mice were purchased from Charles River (Wilmington, MA) and maintained in a 12-h dark/light cycle at 30 lux at the Neuroscience Center of Excellence, Louisiana State University Health, New Orleans, LA. The animals were handled in compliance with the guidelines of the Association for Research in Vision and Ophthalmology Statement for the Use of Animals in Ophthalmic and Vision Research, and the experimental protocols were approved by the Institutional Animal Care and Use Committee at Louisiana State University Health, New Orleans.

### Injury and treatments

Mice were anesthetized by an i.p. injection of ketamine (200 mg/kg) and xylazine (10 mg/kg). Drops of proparacaine were used as topical anesthesia. The mouse corneas (right eye) were injured by rotating a 2-mm diameter trephine in the central area of the cornea at the level of epithelium and one-third of the anterior stroma, allowing the stromal nerves to be damaged. All surgeries were performed by the same investigator (J. H.). After injury, corneas were treated topically with 10 μl of the compounds dissolved in PBS as described in Table 1. In some experiments, mice were injected i.p. with the PEDF-R inhibitor, atglistatin, 1 h before injury, and then atglistatin was applied topically before topical treatment with PEDF + DHA. For i.p. administration, atglistatin was prepared as described previously (38), adjusted to pH 7 with a Tris base, and dissolved in PBS containing 0.25% Cremophor® EL (Millipore, Sigma) (38). All procedures are summarized in the experimental schemes of Figs. 2, 3, 5, and 6.

### Measurement of tear volume (Schirmer's test)

Tears were assessed (without anesthesia) with a phenol red-soaked cotton thread (Menicon America Inc., San Mateo, CA) applied to the lateral canthus for 15 s. The wetting length of the thread was read by an examiner (T. L. P.) in a masked fashion under a microscope by using a ruler offered by the manufacturer (41). Tear production was measured on days 2, 4, and 6 after injury before starting treatments for the day.

### Tear and tissue sample preparation

For tear collection, 5 μl of PBS was applied to the ocular surface for 30 s and then collected from the tear meniscus in the lateral canthus. Samples from six eyes were pooled. All samples were collected by the same researcher (T. L. P.) and kept at −80 °C until Western blot analysis was performed. For qPCR analysis, the mice were euthanized, and corneas and TG (six per sample) were harvested and kept in RNAlater solution (Ambion, Austin, TX) for storage without jeopardizing RNA quality or quantity. For immunoblot and lipidomic analyses, the TG and corneas were dissected and snap-frozen in liquid nitrogen. Tissues were homogenized in lysis buffer containing a mixture of protease and phosphatase inhibitors (Millipore, Sigma). Protein concentrations were measured using protein assay kit I from Bio-Rad.

### Cornea ex vivo organ culture

The mice were euthanized, and injured corneas were dissected and placed into a 12-well plate with the epithelium facing up. A pool of six corneas/well were cultured in 1 ml of DMEM/F-12 medium supplemented with 5% penicillin/streptomycin (Thermo Fisher Scientific) containing full-length PEDF + DHA, NPD1, or the 44-mer (Val-78–Thr-121) neuroprotective domain of PEDF + DHA at the concentrations described in Table 1. After culturing for 24 h at 37 °C (5% CO_2_), the media were collected, centrifuged at 13,000 rpm/5 min at 4 °C to remove cell debris, and precipitated with trichloroacetic acid (TCA). Because a small amount of protein could be lost in the precipitation, 200 ng of GAPDH protein (Abcam, MA) was added as an internal standard before precipitating. The pellet containing secreted proteins was resuspended and denatured with 80 μl of 1× NuPAGE LDS sample buffer (Thermo Fisher Scientific) at 85 °C for 5 min and then analyzed by Western blotting.

### Gene expression analysis

Corneas and TG samples in RNAlater solution were washed with PBS, dried using a paper towel, and then kept in RLT lysis buffer (Qiagen, Germany). All samples were homogenized on ice with a Dounce homogenizer. Total mRNA was extracted using an RNeasy mini kit (Qiagen) as described by the manufacturer. Purity and concentration of RNA were determined with a NanoDrop ND-1000 spectrophotometer (Thermo Fisher Scientific), although the RNA integrity was analyzed using agarose gel electrophoresis. For spectrophotometer analysis, an *A*_260_/*A*_280_ ratio between 1.8 and 2.0 was considered acceptable. For electrophoresis, the 28S and 18S ribosomal RNA bands were sharp and intense, and the density ratio of the 28S/18S was higher than 2. RNA samples were stored at −80 °C until they were used. For quantitative real time PCR, 1 μg of RNA was reverse-transcribed using iScript Reverse Transcription Supermix (Bio-Rad), and the cDNA was quantified using SsoAdvance Universal SYBR Green Supermix (Bio-Rad). Data were collected using CFX384 real-time PCR detection system (Bio-Rad) and analyzed using CFX Manager 3.0 software (Bio-Rad) by the ΔΔ*Ct* method. For the first screening of gene expression, 62 genes were analyzed by using a custom-made Prime PCR panel for the neurotrophins and receptors (SAB Target List, M384 Predesigned 384-well panel) (Bio-Rad); all the primers were validated. For further analysis, primers were designed, and their sequences are described in Table 2. The primers were synthesized by Eurofins MWG Operon LLC.

### Immunostaining and imaging

To study nerve density, whole-mount staining was performed. Corneas were fixed and stained with rabbit anti-PGP9.5 (Abcam, 1:1000) and rat anti-SP (Santa Cruz Biotechnology, 1:200) monoclonal antibodies as described previously (20). Pictures were taken with a fluorescent microscope (Olympus IX71; Olympus Corp., Tokyo, Japan). For the whole-mount view, the images were taken with ×10 objective lens. All images at the same layer recorded from one cornea were merged together to build an entire view of the corneal nerve network with Photoshop CC 2014 (Adobe). For the best illustration of corneal nerves, the merged images were changed to black-and-white mode with the black background and then inverted for the white background (Fig. 1*C*). The corneal nerve densities were measured by Photoshop CC 2014 (Adobe).

For corneal tissue section staining, the eyeballs were enucleated and fixed in freshly prepared 2% paraformaldehyde for 1 h at room temperature, and then they were embedded in optimal cutting temperature (OCT) compound. Eight-μm serial sections were washed with PBS (three times for 5 min), blocked with 10% normal goat serum plus 0.5% Triton X-100 solution in PBS for 1 h, and then incubated at 4 °C overnight with primary rabbit anti-PEDF-R antibody (Cayman, Ann Arbor, MI; 1:250). After washing with PBS (three times for 5 min), the sections were incubated with Alexa Fluor 488 goat anti-rabbit IG (H+L) secondary antibody (Thermo Fisher Scientific) for 1 h at room temperature. The specificity of the antibody was assessed in controls in which the primary antibody was replaced with the same host IgG. No staining was found.

### Western blotting

For the tissues, 50 μg of total protein was subjected to SDS-PAGE using Novex 4–12% BisTris gels (Thermo Fisher Scientific) and transferred to 0.2 μm PVDF membranes (Bio-Rad). Nonspecific binding was blocked with 5% nonfat dry milk (Bio-Rad) in PBS with 1% Tween 20 (PBST) for 1 h at room temperature. After washing with PBST (three times for 5 min), the membranes were incubated with the primary antibodies (at 4 °C, overnight) followed by washing with PBST (three times for 5 min), and then the membranes were incubated with the corresponding secondary antibodies for 1 h at room temperature. Information on the antibodies used in this study is provided in Table 3. Protein bands were visualized using chemiluminescence detection reagents (Thermo Fisher Scientific), and the intensity of immunoreactive bands was quantified using an LAS 4000 imaging system (GE Healthcare). For tear samples, 7 μg of the protein collected from the mouse tear film (pool of six eyes) was used.

### Lipid extraction and LC-MS/MS-based lipidomic analysis

Cornea samples were homogenized in 3 ml of MeOH followed by the addition of 6 ml of CHCl_3_ and 5 μl of an internal standard mixture of deuterium-labeled lipids (AA-*d*_8_ (5 ng/μl), PGD2-*d*_4_ (1 ng/μl), EPA-*d*_5_ (1 ng/μl), 15-HETE-*d*_8_ (1 ng/μl), and LTB4-*d*_4_ (1 ng/μl)). The samples were sonicated for 30 min and stored at −80 °C overnight. The supernatant was collected, and the pellet was washed with 1 ml of CHCl_3_/MeOH (2:1) and centrifuged, and then the supernatants were combined. Two ml of distilled water, pH 3.5, was added to the supernatant, vortexed, and centrifuged, and then the pH of the upper phase was adjusted to 3.5–4.0 with 0.1 n HCl. The lower phase was dried down under N_2_ and then resuspended in 1 ml of MeOH.

LC-MS/MS analysis was performed in a Xevo TQ equipped with Acquity I class UPLC with a flow-through needle (Waters). For PC and PE molecular species analysis, the samples were dried under N_2_ and then resuspended in 20 μl of the sample solvent (acetonitrile/chloroform/methanol, 90:5:5 by volume). The Acquity UPLC BEH HILIC 1.7-μm, 2.1 × 100-mm column was used with a mixture of solvent A (acetonitrile/water, 1:1; 10 mm ammonium acetate, pH 8.3) and solvent B (acetonitrile/water, 95:5; 10 mm ammonium acetate, pH 8.3) as the mobile phase, which was flowing at a rate at 0.5 ml/min. Solvent B (100%) was isocratically run for the first 5 min and then run in a gradient to 20% of solvent A for 8 min, increased to 65% of solvent A for 0.5 min, run isocratically at 65% of solvent A for 3 min, and then returned to 100% of solvent B for 3.5 min for equilibration. The column temperature was set to 30 °C. The amount for each PC and PE species was calculated as the percentage of the total PCs and PEs in each cornea sample.

For analysis of fatty acids and their derivatives, six corneas were pooled and homogenized as described above. Samples (in 1 ml of MeOH) were mixed with 9 ml of H_2_O at pH 3.5, loaded onto C18 columns (Agilent, CA), and then eluted with 10 ml of methyl formate. Samples were dried under N_2_, resuspended in 20 μl of MeOH/H_2_O (2:1), and injected into an Acquity UPLC HSS T3 1.8-μm 2.1 × 50-mm column. The mobile phase consisted of 45% solvent A (H_2_O + 0.01% acetic acid) and 55% solvent B (MeOH + 0.01% acetic acid), with a 0.4 ml/min flow used initially, and then a gradient to 15% solvent A for the first 10 min, a gradient to 2% solvent A for 18 min, 2% solvent A run isocratically until 25 min, and then a gradient back to 45% solvent A for re-equilibration until 30 min. Lipid standards (Cayman, Ann Arbor, MI) were used for tuning and optimization, as well as to create calibration curves for each compound.

### Statistics

Data were expressed as the mean ± S.D. of two or more independent experiments. Statistical comparisons were performed using Minitab 17 software (Minitab Inc.), using Student's *t* test or one-way ANOVA followed by Fisher post hoc test at 95% confidence levels. *p* values of <0.05 were considered significant. Graphs were made using GraphPad Prism 7 software (GraphPad Software).

## Author contributions

H. E. P. B. and T. L. P. contributed to designing the experiments. T. L. P. contributed to the acquisition and analysis of data, drafting, and critical review of the manuscript. J. H. performed the surgery and extraction of tissues and helped with the immunohistochemistry. A. H. K. prepared the drugs, performed RNA extractions and assays of tear volume, and helped with qPCR. B. J. performed the LC-MS/MS lipid analysis and acquisition of data. J. H., A. H. K., and B. J. provided critical review of the manuscript. N. G. B. contributed to interpretation of results and reviewed the manuscript. H. E. P. B. supervised the study, wrote and reviewed the manuscript, and was responsible for the integrity of this work. H. E. P. B. is the guarantor of this work and, as such, has full access to all data in the study and takes responsibility for the integrity of the data and the accuracy of the data analysis.

## Supplementary Material

Supplemental Data

## Footnotes

2 The abbreviations used are:

LASIK: laser *in situ* keratomileusis
AA: arachidonic acid
DHA: docosahexaenoic acid
GPI: glycophosphatidylinositol
HDHA: hydroxyl-DHA
iPLA_2_: calcium-independent phospholipase A_2_
NPD1: neuroprotectin D1
PC: phosphatidylcholine
PE: phosphatidylethanolamine
PEDF: pigment epithelium–derived factor
PEDF-R: PEDF receptor
SP: substance P
RAG: regeneration-associated gene
qPCR: quantitative PCR
TG: trigeminal ganglia
BisTris: 2-[bis(2-hydroxyethyl)amino]-2-(hydroxymethyl)propane-1,3-diol
HETE: hydroxyeicosatetraenoic acid
CGRP: calcitonin gene-related peptide
Trk: tropomyosin receptor kinase
ANOVA: analysis of variance
HpDHA: (*S*)-hydroperoxy-DHA.

## References

[1] Shaheen B. S., Bakir M., and Jain S. (2014) Corneal nerves in health and disease. Surv. Ophthalmol. 59, 263–285 24461367 10.1016/j.survophthal.2013.09.002 PMC4004679

[2] Müller L. J., Marfurt C. F., Kruse F., and Tervo T. M. (2003) Corneal nerves: structure, contents and function. Exp. Eye Res. 76, 521–542 12697417 10.1016/s0014-4835(03)00050-2

[3] Chao C., Golebiowski B., and Stapleton F. (2014) The role of corneal innervation in LASIK-induced neuropathic dry eye. Ocul. Surf. 12, 32–45 24439045 10.1016/j.jtos.2013.09.001

[4] Erie J. C., McLaren J. W., Hodge D. O., and Bourne W. M. (2005) Recovery of corneal subbasal nerve density after PRK and LASIK. Am. J. Ophthalmol. 140, 1059–1064 16376651 10.1016/j.ajo.2005.07.027

[5] Kymionis G. D., Tsiklis N., Pallikaris A. I., Bouzoukis D. I., and Pallikaris I. G. (2007) Fifteen-year follow-up after LASIK: case report. J. Refract. Surg. 23, 937–940 18041250 10.3928/1081-597X-20071101-13

[6] Tombran-Tink J., and Barnstable C. J. (2003) PEDF: a multifaceted neurotrophic factor. Nat. Rev. Neurosci. 4, 628–636 12894238 10.1038/nrn1176

[7] Subramanian P., Locatelli-Hoops S., Kenealey J., DesJardin J., Notari L., and Becerra S. P. (2013) Pigment epithelium-derived factor (PEDF) prevents retinal cell death via PEDF receptor (PEDF-R) identification of a functional ligand binding site. J. Biol. Chem. 288, 23928–23942 23818523 10.1074/jbc.M113.487884 PMC3745339

[8] Bilak M. M., Becerra S. P., Vincent A. M., Moss B. H., Aymerich M. S., and Kuncl R. W. (2002) Identification of the neuroprotective molecular region of pigment epithelium-derived factor and its binding sites on motor neurons. J. Neurosci. 22, 9378–9386 12417663 10.1523/JNEUROSCI.22-21-09378.2002 PMC6758058

[9] He J., Cortina M. S., Kakazu A., and Bazan H. E. (2015) The PEDF neuroprotective domain plus DHA induces corneal nerve regeneration after experimental surgery. Invest. Ophthalmol. Vis. Sci. 56, 3505–3513 26030104 10.1167/iovs.15-16755 PMC4463800

[10] Cortina M. S., He J., Li N., Bazan N. G., and Bazan H. E. (2010) Neuroprotectin D1 synthesis and corneal nerve regeneration after experimental surgery and treatment with PEDF plus DHA. Invest. Ophthalmol. Vis. Sci. 51, 804–810 19797230 10.1167/iovs.09-3641 PMC2868452

[11] Cortina M. S., He J., Li N., Bazan N. G., and Bazan H. E. (2012) Recovery of corneal sensitivity, calcitonin gene-related peptide-positive nerves, and increased wound healing induced by pigment epithelial-derived factor plus docosahexaenoic acid after experimental surgery. Arch. Ophthalmol. 130, 76–83 21911652 10.1001/archophthalmol.2011.287

[12] Cortina M. S., He J., Russ T., Bazan N. G., and Bazan H. E. (2013) Neuroprotectin D1 restores corneal nerve integrity and function after damage from experimental surgery. Invest. Ophthalmol. Vis. Sci. 54, 4109–4116 23702780 10.1167/iovs.13-12075 PMC3681478

[13] Notari L., Baladron V., Aroca-Aguilar J. D., Balko N., Heredia R., Meyer C., Notario P. M., Saravanamuthu S., Nueda M.-L., Sanchez-Sanchez F., Escribano J., Laborda J., and Becerra S. P. (2006) Identification of a lipase-linked cell membrane receptor for pigment epithelium-derived factor. J. Biol. Chem. 281, 38022–38037 17032652 10.1074/jbc.M600353200

[14] Jenkins C. M., Mancuso D. J., Yan W., Sims H. F., Gibson B., and Gross R. W. (2004) Identification, cloning, expression, and purification of three novel human calcium-independent phospholipase A2 family members possessing triacylglycerol lipase and acylglycerol transacylase activities. J. Biol. Chem. 279, 48968–48975 15364929 10.1074/jbc.M407841200

[15] Zimmermann R., Strauss J. G., Haemmerle G., Schoiswohl G., Birner-Gruenberger R., Riederer M., Lass A., Neuberger G., Eisenhaber F., Hermetter A., and Zechner R. (2004) Fat mobilization in adipose tissue is promoted by adipose triglyceride lipase. Science 306, 1383–1386 15550674 10.1126/science.1100747

[16] Villena J. A., Roy S., Sarkadi-Nagy E., Kim K.-H., and Sul H. S. (2004) Desnutrin, an adipocyte gene encoding a novel patatin domain-containing protein, is induced by fasting and glucocorticoids: ectopic expression of desnutrin increases triglyceride hydrolysis. J. Biol. Chem. 279, 47066–47075 15337759 10.1074/jbc.M403855200

[17] Bartness T. J., Liu Y., Shrestha Y. B., and Ryu V. (2014) Neural innervation of white adipose tissue and the control of lipolysis. Front. Neuroendocrinol. 35, 473–493 24736043 10.1016/j.yfrne.2014.04.001 PMC4175185

[18] Bartness T. J., Vaughan C. H., and Song C. K. (2010) Sympathetic and sensory innervation of brown adipose tissue. Int. J. Obes. 34, 36–42 10.1038/ijo.2010.182 PMC3999344 20935665

[19] He J., Bazan N. G., and Bazan H. E. (2010) Mapping the entire human corneal nerve architecture. Exp. Eye Res. 91, 513–523 20650270 10.1016/j.exer.2010.07.007 PMC2939211

[20] He J., and Bazan H. E. (2016) Neuroanatomy and neurochemistry of mouse cornea. Invest. Ophthalmol. Vis. Sci. 57, 664–674 26906155 10.1167/iovs.15-18019 PMC4771196

[21] Ferrari G., Bignami F., Giacomini C., Capitolo E., Comi G., Chaabane L., and Rama P. (2014) Ocular surface injury induces inflammation in the brain: *in vivo* and *ex vivo* evidence of a corneal–trigeminal axis. Invest. Ophthalmol. Vis. Sci. 55, 6289–6300 25146993 10.1167/iovs.14-13984

[22] Lindsay R. M. (1988) Nerve growth factors (NGF, BDNF) enhance axonal regeneration but are not required for survival of adult sensory neurons. J. Neurosci. 8, 2394–2405 3249232 10.1523/JNEUROSCI.08-07-02394.1988 PMC6569525

[23] Pasterkamp R. J., Peschon J. J., Spriggs M. K., and Kolodkin A. L. (2003) Semaphorin 7A promotes axon outgrowth through integrins and MAPKs. Nature 424, 398–405 12879062 10.1038/nature01790

[24] Finelli M. J., Wong J. K., and Zou H. (2013) Epigenetic regulation of sensory axon regeneration after spinal cord injury. J. Neurosci. 33, 19664–19676 24336730 10.1523/JNEUROSCI.0589-13.2013 PMC3858634

[25] Mulderry P. K., and Dobson S. P. (1996) Regulation of VIP and other neuropeptides by c-Jun in sensory neurons: implications for the neuropeptide response to axotomy. Eur. J. Neurosci. 8, 2479–2491 8996797 10.1111/j.1460-9568.1996.tb01542.x

[26] Starkey M. L., Davies M., Yip P. K., Carter L. M., Wong D. J., McMahon S. B., and Bradbury E. J. (2009) Expression of the regeneration-associated protein SPRR1A in primary sensory neurons and spinal cord of the adult mouse following peripheral and central injury. J. Comp. Neurol. 513, 51–68 19107756 10.1002/cne.21944 PMC3339437

[27] Chaudhary S., Namavari A., Yco L., Chang J.-H., Sonawane S., Khanolkar V., Sarkar J., and Jain S. (2012) Neurotrophins and nerve regeneration-associated genes are expressed in the cornea after lamellar flap surgery. Cornea 31, 1460–1467 22673847 10.1097/ICO.0b013e318247b60e PMC3612527

[28] You L., Kruse F. E., and Völcker H. E. (2000) Neurotrophic factors in the human cornea. Invest. Ophthalmol. Vis. Sci. 41, 692–702 10711683

[29] Chang H.-M., Shyu M.-K., Tseng G.-F., Liu C.-H., Chang H.-S., Lan C.-T., Hsu W.-M., and Liao W.-C. (2013) Neuregulin facilitates nerve regeneration by speeding Schwann cell migration via ErbB2/3-dependent FAK pathway. PLoS ONE 8, e53444 23301073 10.1371/journal.pone.0053444 PMC3534691

[30] Grothe C., and Nikkhah G. (2001) The role of basic fibroblast growth factor in peripheral nerve regeneration. Anat. Embryol. 204, 171–177 11681796 10.1007/s004290100205

[31] Seidah N. G., Benjannet S., Pareek S., Savaria D., Hamelin J., Goulet B., Laliberte J., Lazure C., Chrétien M., and Murphy R. A. (1996) Cellular processing of the nerve growth factor precursor by the mammalian pro-protein convertases. Biochem. J. 314, 951–960 8615794 10.1042/bj3140951 PMC1217149

[32] Martin-Zanca D., Oskam R., Mitra G., Copeland T., and Barbacid M. (1989) Molecular and biochemical characterization of the human trk proto-oncogene. Mol. Cell. Biol. 9, 24–33 2927393 10.1128/mcb.9.1.24-33.1989 PMC362141

[33] Du J., Feng L., Zaitsev E., Je H.-S., Liu X. W., and Lu B. (2003) Regulation of TrkB receptor tyrosine kinase and its internalization by neuronal activity and Ca^2+^ influx. J. Cell Biol. 163, 385–395 14581459 10.1083/jcb.200305134 PMC2173520

[34] Eixarch H., Gutiérrez-Franco A., Montalban X., and Espejo C. (2013) Semaphorins 3A and 7A: potential immune and neuroregenerative targets in multiple sclerosis. Trends Mol. Med. 19, 157–164 23419749 10.1016/j.molmed.2013.01.003

[35] Namavari A., Chaudhary S., Ozturk O., Chang J.-H., Yco L., Sonawane S., Katam N., Khanolkar V., Hallak J., Sarkar J., and Jain S. (2012) Semaphorin 7a links nerve regeneration and inflammation in the cornea. Invest. Ophthalmol. Vis. Sci. 53, 4575–4585 22700709 10.1167/iovs.12-9760 PMC3394693

[36] Bazan H. E., and Bazan N. G. (1984) Composition of phospholipids and free fatty acids and incorporation of labeled arachidonic acid in rabbit cornea. Comparison of epithelium, stroma and endothelium. Curr. Eye Res. 3, 1313–1319 6439475 10.3109/02713688409007418

[37] Crane A. M., Hua H.-U., Coggin A. D., Gugiu B. G., Lam B. L., and Bhattacharya S. K. (2012) Mass spectrometric analyses of phosphatidylcholines in alkali-exposed corneal tissue. Invest. Ophthalmol. Vis. Sci. 53, 7122–7130 22956606 10.1167/iovs.12-10448 PMC3487488

[38] Mayer N., Schweiger M., Romauch M., Grabner G. F., Eichmann T. O., Fuchs E., Ivkovic J., Heier C., Mrak I., Lass A., Höfler G., Fledelius C., Zechner R., Zimmermann R., and Breinbauer R. (2013) Development of small-molecule inhibitors targeting adipose triglyceride lipase. Nat. Chem. Biol. 9, 785–787 24096302 10.1038/nchembio.1359 PMC3829776

[39] Bazan N. G. (2009) Neuroprotectin D1-mediated anti-inflammatory and survival signaling in stroke, retinal degenerations, and Alzheimer's disease. J. Lipid Res. 50, S400–S405 19018037 10.1194/jlr.R800068-JLR200 PMC2674685

[40] Serhan C. N., Dalli J., Colas R. A., Winkler J. W., and Chiang N. (2015) Protectins and Maresins: new pro-resolving families of mediators in acute inflammation and resolution bioactive metabolome. Biochim. Biophys. Acta 1851, 397–413 25139562 10.1016/j.bbalip.2014.08.006 PMC4324013

[41] Li N., He J., Schwartz C. E., Gjorstrup P., and Bazan H. E. (2010) Resolvin E1 improves tear production and decreases inflammation in a dry eye mouse model. J. Ocul. Pharmacol. Ther. 26, 431–439 20874497 10.1089/jop.2010.0019 PMC2956380

[42] He J., Pham T. L., Kakazu A., and Bazan H. E. (2017) Recovery of corneal sensitivity and increase in nerve density and wound healing in diabetic mice after PEDF plus DHA treatment. Diabetes 66, 2511–2520 28592408 10.2337/db17-0249 PMC5566302

[43] MacLean D. B. (1987) Substance P synthesis and transport in explants of nodose ganglion/vagus nerve: effects of double ligation, 2-deoxyglucose, veratridine, and ouabain. J. Neurochem. 48, 1794–1803 2437249 10.1111/j.1471-4159.1987.tb05738.x

[44] Amadesi S., Reni C., Katare R., Meloni M., Oikawa A., Beltrami A. P., Avolio E., Cesselli D., Fortunato O., Spinetti G., Ascione R., Cangiano E., Valgimigli M., Hunt S. P., Emanueli C., and Madeddu P. (2012) Role for substance P-based nociceptive signaling in progenitor cell activation and angiogenesis during ischemia in mice and in human subjects. Circulation 125, 1774–1786 22392530 10.1161/CIRCULATIONAHA.111.089763 PMC3616366

[45] Esquenazi S., Bazan H. E., Bui V., He J., Kim D. B., and Bazan N. G. (2005) Topical combination of NGF and DHA increases rabbit corneal nerve regeneration after photorefractive keratectomy. Invest. Ophthalmol. Vis. Sci. 46, 3121–3127 16123410 10.1167/iovs.05-0241

[46] Yabe T., Wilson D., and Schwartz J. P. (2001) NFκB activation is required for the neuroprotective effects of pigment epithelium-derived factor (PEDF) on cerebellar granule neurons. J. Biol. Chem. 276, 43313–43319 11553640 10.1074/jbc.M107831200

[47] Barlati S., Marchina E., Quaranta C. A., Vigasio F., and Semeraro F. (1990) Analysis of fibronectin, plasminogen activators and plasminogen in tear fluid as markers of corneal damage and repair. Exp. Eye Res. 51, 1–9 2115456 10.1016/0014-4835(90)90162-n

[48] Salton S., and Lin W. J. (2013) The regulated secretory pathway and human disease: insights from gene variants and single nucleotide polymorphisms. Front. Endocrinol. 4, 96 10.3389/fendo.2013.00096 PMC3734370 23964269

[49] Yamada A., Kubo K., Takeshita T., Harashima N., Kawano K., Mine T., Sagawa K., Sugamura K., and Itoh K. (1999) Molecular cloning of a glycosylphosphatidylinositol-anchored molecule CDw108. J. Immunol. 162, 4094–4100 10201933

[50] Campbell D., Griffiths G., and Tighe B. J. (2011) Tear analysis and lens–tear interactions: part II. Ocular lipids-nature and fate of meibomian gland phospholipids. Cornea 30, 325–332 21304291 10.1097/ICO.0b013e3181eae239

[51] Leppä S., Saffrich R., Ansorge W., and Bohmann D. (1998) Differential regulation of c-Jun by ERK and JNK during PC12 cell differentiation. EMBO J. 17, 4404–4413 9687508 10.1093/emboj/17.15.4404 PMC1170773

[52] Chung J., Uchida E., Grammer T. C., and Blenis J. (1997) STAT3 serine phosphorylation by ERK-dependent and -independent pathways negatively modulates its tyrosine phosphorylation. Mol. Cell. Biol. 17, 6508–6516 9343414 10.1128/mcb.17.11.6508 PMC232504

[53] Haemmerle G., Lass A., Zimmermann R., Gorkiewicz G., Meyer C., Rozman J., Heldmaier G., Maier R., Theussl C., Eder S., Kratky D., Wagner E. F., Klingenspor M., Hoefler G., and Zechner R. (2006) Defective lipolysis and altered energy metabolism in mice lacking adipose triglyceride lipase. Science 312, 734–737 16675698 10.1126/science.1123965

[54] van Meer G., Voelker D. R., and Feigenson G. W. (2008) Membrane lipids: where they are and how they behave. Nat. Rev. Mol. Cell Biol. 9, 112–124 18216768 10.1038/nrm2330 PMC2642958

[55] Gronert K., Maheshwari N., Khan N., Hassan I. R., Dunn M., and Laniado Schwartzman M. (2005) A role for the mouse 12/15-lipoxygenase pathway in promoting epithelial wound healing and host defense. J. Biol. Chem. 280, 15267–15278 15708862 10.1074/jbc.M410638200

