# Supplementary material for: Defining a mechanistic link between pigment epithelium–derived factor, docosahexaenoic acid, and corneal nerve regeneration
Source: J Biol Chem. 2017 Sep 26;292(45):18486–99. doi: 10.1074/jbc.M117.801472 (PMC5682960; doi:10.1074/jbc.M117.801472)
Supplement: Supplemental Data [file supp_292_45_18486__index.html]

Defining a mechanistic link between pigment epithelium-derived factor, docosahexaenoic acid and corneal nerve regeneration — Defining a mechanistic link between pigment epithelium–derived factor, docosahexaenoic acid, and corneal nerve regeneration — PEDF + DHA signaling stimulates corneal nerve regeneration — Supplemental Data 

# Defining a mechanistic link between pigment epithelium–derived factor, docosahexaenoic acid, and corneal nerve regeneration

## Supplemental Data

- Supplemental info (.docx, 196 KB) - Supplemental info
