# Supplementary material for: Defining a mechanistic link between pigment epithelium–derived factor, docosahexaenoic acid, and corneal nerve regeneration
Source: J Biol Chem. 2017 Sep 26;292(45):18486–99. doi: 10.1074/jbc.M117.801472 (PMC5682960; doi:10.1074/jbc.M117.801472)
Supplement: Supplemental Data [file 10.1074_M117.801472_jbc.M117.801472-1.docx]

**Defining a mechanistic link between pigment epithelium-derived factor, docosahexaenoic acid and corneal nerve regeneration**

Thang Luong Pham, Jiucheng He, Azucena Kakazu, Bokkyoo Jun, Nicolas G. Bazan, Haydee E. P. Bazan

Department of Ophthalmology and Neuroscience Center of Excellence, School of Medicine, Louisiana State University Health New Orleans, New Orleans, LA, 70112-2223

Supplemental data included:

1. Figure S1: The effect of PEDF+DHA treatment on the phosphorylation of TrkB and ERK1/2 in TG at 6 and 12 hours.
2. Table S1: The list of genes and status for the screening of gene induction by PEDF+DHA using the M384 Predesigned 384-well panel.

**
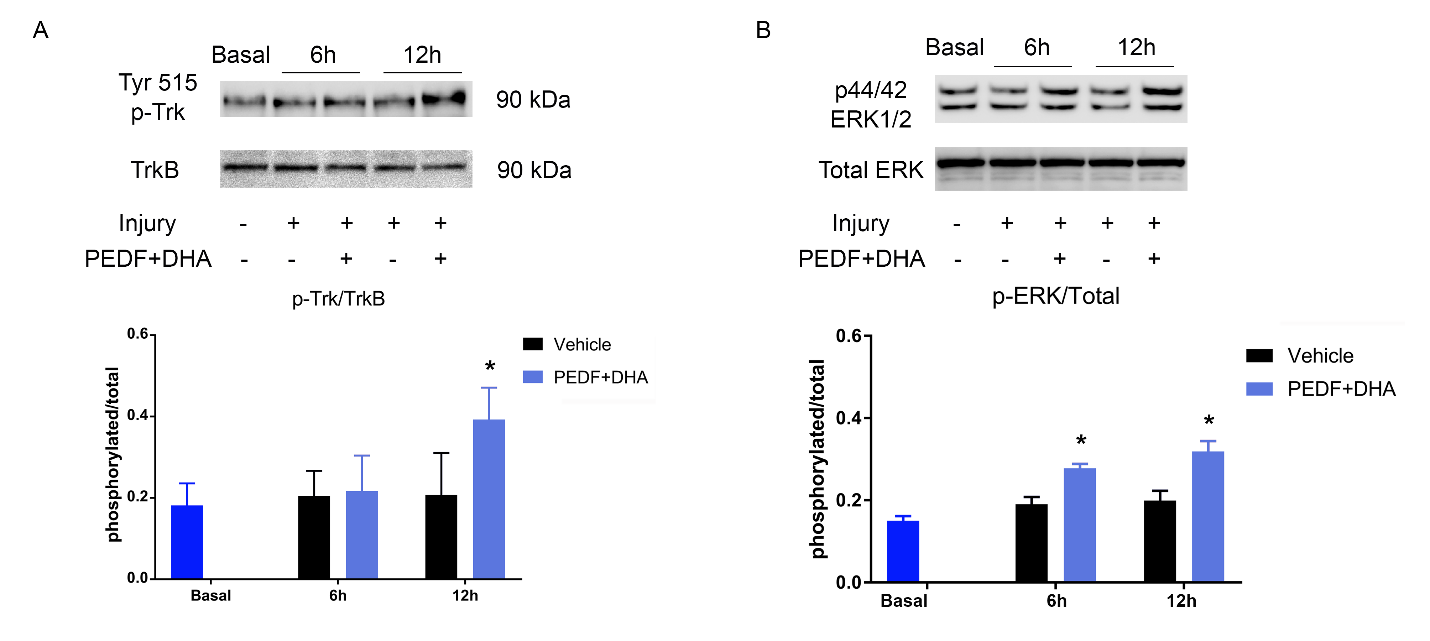
Figure S1: Effect of** **PEDF+DHA treatment on the phosphorylation of TrkB and ERK1/2 in TG at 6 and 12 hours**. (A) Western blot analysis of Tyr-phosphorylated TrkB and total TrkB in the TG (pool of six TGs, 50 µg protein per well) after treatment with PEDF+DHA for 6 and 12 hours. (B) Western blot analysis of p44/p42 ERK 1/2 and total ERK in the TG (pool of six TGs, 50 µg protein per well). Bars represent the mean of three samples ± SD. *p < 0.05 with t-test analysis in comparison to the vehicle at the same time points.

**Table S1:** The list of genes and status for the screening of gene induction by PEDF+DHA using the M384 Predesigned 384-well panel.

| No. | Gene | Name | Status |
| --- | --- | --- | --- |
| 1 | Bdnf | brain-derived neurotrophic factor | Up-regulated |
| 2 | Cntfr | ciliary neurotrophic factor receptor | Up-regulated |
| 3 | Fgf2 | fibroblast growth factor 2 | Up-regulated |
| 4 | Gdnf | glial cell derived neurotrophic factor | Up-regulated |
| 5 | Ngf | nerve growth factor (beta polypeptide) | Up-regulated |
| 6 | Gmfg | glia maturation factor, gamma | Down-regulated |
| 7 | Maged1 | melanoma antigen family D, 1 | Down-regulated |
| 8 | Ngfrap1 | nerve growth factor receptor (TNFRSF16) associated protein 1 | Down-regulated |
| 9 | Ntf5 | neurotrophin 5 | Down-regulated |
| 10 | Sema3A | semaphorin 3A | Down-regulated |
| 11 | Adcyap1r1 | Adenylate Cyclase Activating Polypeptide 1 (Pituitary) Receptor Type I | Unchanged |
| 12 | Artn | artemin | Unchanged |
| 13 | Cbln1 | cerebellin 1 precursor | Unchanged |
| 14 | Cntf | ciliary neurotrophic factor | Unchanged |
| 15 | Crhbp | corticotropin releasing hormone binding protein | Unchanged |
| 16 | Frs2 | fibroblast growth factor receptor substrate 2 | Unchanged |
| 17 | Frs3 | fibroblast growth factor receptor substrate 3 | Unchanged |
| 18 | Fus | fused in sarcoma | Unchanged |
| 19 | Galr2 | galanin receptor 2 | Unchanged |
| 20 | Gfra1 | GDNF family receptor alpha 1 | Unchanged |
| 21 | Gfra2 | GDNF family receptor alpha 2 | Unchanged |
| 22 | Gfra3 | GDNF family receptor alpha 3 | Unchanged |
| 23 | Gmfb | glia maturation factor, beta | Unchanged |
| 24 | Mef2c | myocyte enhancer factor 2C | Unchanged |
| 25 | Mt3 | metallothionein 3 | Unchanged |
| 26 | Nell1 | NEL-like 1 (chicken) | Unchanged |
| 27 | Nf1 | neurofibromin 1 | Unchanged |
| 28 | Ngfr | nerve growth factor receptor | Unchanged |
| 29 | Npff | neuropeptide FF-amide peptide precursor | Unchanged |
| 30 | Npy1r | neuropeptide Y receptor Y1 | Unchanged |
| 31 | Nr1i2 | nuclear receptor subfamily 1, group I, member 2 | Unchanged |
| 32 | Nrg1 | neuregulin 1 | Unchanged |
| 33 | Nrg2 | neuregulin 2 | Unchanged |
| 34 | Ntf3 | neurotrophin 3 | Unchanged |
| 35 | Ntrk1 | neurotrophic tyrosine kinase, receptor, type 1 | Unchanged |
| 36 | Ntrk2 | neurotrophic tyrosine kinase, receptor, type 2 | Unchanged |
| 37 | Ppyr1 | neuropeptide Y receptor Y4 | Unchanged |
| 38 | Pspn | persephin | Unchanged |
| 39 | Ptger2 | prostaglandin E receptor 2 (subtype EP2), 53kDa | Unchanged |
| 40 | Stat3 | signal transducer and activator of transcription 3 (acute-phase response factor) | Unchanged |
| 41 | tac1 | Tachykinin Precursor 1 | Unchanged |
| 42 | Tacr1 | tachykinin receptor 1 | Unchanged |
| 43 | Tfg | TRK-fused gene | Unchanged |
| 44 | Vgf | VGF nerve growth factor inducible | Unchanged |
| 45 | Cckar | cholecystokinin A receptor | Not detected |
| 46 | Crh | corticotropin releasing hormone | Not detected |
| 47 | Crhr1 | corticotropin releasing hormone receptor 1 | Not detected |
| 48 | Crhr2 | corticotropin releasing hormone receptor 2 | Not detected |
| 49 | Gal | galanin/GMAP prepropeptide | Not detected |
| 50 | Galr1 | galanin receptor 1 | Not detected |
| 51 | Grpr | gastrin-releasing peptide receptor | Not detected |
| 52 | Hcrt | hypocretin (orexin) neuropeptide precursor | Not detected |
| 53 | Mc2r | melanocortin 2 receptor (adrenocorticotropic hormone) | Not detected |
| 54 | Npffr2 | neuropeptide FF receptor 2 | Not detected |
| 55 | Npy2r | neuropeptide Y receptor Y2 | Not detected |
| 56 | Ntsr1 | neurotensin receptor 1 (high affinity) | Not detected |
| 57 | Pnoc | prepronociceptin | Not detected |
| 58 | Ucn | urocortin | Not detected |
| 59 | Tfrc | transferrin receptor | Not detected |
| 60 | Tbp | TATA box binding protein | Not detected |
| 61 | Actb | actin, beta | Not detected |
| 62 | Gapdh | glyceraldehyde-3-phosphate dehydrogenase | Not detected |
